# Abnormal Baseline Brain Activity in Patients with Pulsatile Tinnitus: A Resting-State fMRI Study

**DOI:** 10.1155/2014/549162

**Published:** 2014-04-24

**Authors:** Lv Han, Liu Zhaohui, Yan Fei, Li Ting, Zhao Pengfei, Du Wang, Dong Cheng, Guo Pengde, Han Xiaoyi, Wang Xiao, Li Rui, Wang Zhenchang

**Affiliations:** ^1^Department of Radiology Center, Beijing Tongren Hospital, Capital Medical University, Beijing 100730, China; ^2^Department of Radiology Center, Beijing Friendship Hospital, Capital Medical University, Beijing 100050, China

## Abstract

Numerous investigations studying the brain functional activity of the tinnitus patients have indicated that neurological changes are important findings of this kind of disease. However, the pulsatile tinnitus (PT) patients were excluded in previous studies because of the totally different mechanisms of the two subtype tinnitus. The aim of this study is to investigate whether altered baseline brain activity presents in patients with PT using resting-state functional magnetic resonance imaging (rs-fMRI) technique. The present study used unilateral PT patients (*n* = 42) and age-, sex-, and education-matched normal control subjects (*n* = 42) to investigate the changes in structural and amplitude of low-frequency (ALFF) of the brain. Also, we analyzed the relationships between these changes with clinical data of the PT patients. Compared with normal controls, PT patients did not show any structural changes. PT patients showed significant increased ALFF in the bilateral precuneus, and bilateral inferior frontal gyrus (IFG) and decreased ALFF in multiple occipital areas. Moreover, the increased THI score and PT duration was correlated with increased ALFF in precuneus and bilateral IFG. The abnormalities of spontaneous brain activity reflected by ALFF measurements in the absence of structural changes may provide insights into the neural reorganization in PT patients.

## 1. Introduction

Tinnitus is a serious public health problem. 10%–30% of people all over the world are influenced, while 5–26% of them are affected severely [1–5]. Tinnitus can be divided into nonpulsatile or pulsatile subtypes [6]. Most of the patients are nonpulsatile tinnitus (NPT) type. In contrast, pulsatile tinnitus (PT) is relatively rare and has been described in case reports or summaries with a relatively small sample [6–15].

The direct etiology of NPT remains unclear yet. The neurobiological basis may be ongoing abnormal spontaneous neural activity, brain plasticity, or disturbed brain network [16–27], while in contrast, PT is an auditory percept by stimulation of the hair cells in the inner ear. Complaints include a sound like water flow, wind blowing, or beat of the drum in the ear, and so forth. It may be low- to high-pitched tinnitus. Typically, symptomatic improvement could be achieved by external compression of the internal jugular vein in the neck on the symptomatic side. Possible etiologies include sigmoid sinus diverticulum, atherosclerosis, abnormal vascular loops, aneurysm of internal carotid artery, mastoid emissary vein, dural arteriovenous shunts, paraganglioma, involuntary contraction of muscles in the middle ear, and so forth [6–11, 28–36]. The abnormal blood flow induced by a focal defect of mastoid bone shell in the region of the transverse-sigmoid junction is a common etiology according to our daily work. PT is more likely a vascular or muscular originating disorder rather than a true neural activity disorder induced disease.

Increasing attention is being given on the brain functional activity of the tinnitus patients [16–27]. However, these studies were focused only on NPT patients; the PT patients were defined as exclusion criteria because of the totally different mechanisms of the two subtype tinnitus. Thus, it is still not known whether long time PT stimulation can cause the brain abnormalities at present. The totally different etiology between NPT and PT may generate different brain activities. Besides, 20–60% of tinnitus patients have been reported to be depressed [22, 37–41]. The PT patients could also suffer from distress or depression. However, whether there is any corresponding abnormal neural activity remains unclear. An investigation using resting-state functional magnetic resonance imaging (rs-fMRI) technique focusing on brain activities of PT patients is an interesting and essential work.

In this study, we hypothesize that there is altered brain activity in PT patients. These alterations may be considered as neural reorganization or brain plasticity in patients with PT. To the best of our knowledge, there is still no research on the issue.

## 2. Subjects and Methods

### 2.1. Subjects

Forty two patients with persistent unilateral pulsatile tinnitus and 42 healthy controls, matched in gender, age, and right or left handedness, were enrolled in the study. Eighteen patients suffered from left-side pulsatile tinnitus and 24 from right-side pulsatile tinnitus. All of the patients complained of a sound like low- to median-pitched beats of the drum in the ear. Sounds were synchronous with the cardiac activity. Symptomatic improvement could be achieved by external compression of the internal jugular vein in the neck on the symptomatic side. CT angiography (CTA) examinations [42, 43] suspected the etiology of sigmoid sinus diverticulum caused by focal defect of mastoid bone shell in the region of the transverse-sigmoid junction. Digital subtraction angiography (DSA) examinations were also conducted. Other etiologies could be excluded [8]. After surgery, symptoms of all the patients were released. Thus, the etiology of all of the patients was confirmed as a focal defect of mastoid bone shell in the region of the transverse-sigmoid junction. All of the subjects had normal hearing threshold and magnetic resonance imaging findings of brain and had no history of neurological and psychiatric illness, alcohol or drug abuse, and severe visual impairment (the hearing threshold was determined by puretone audiometry (PTA) examination. Participants had hearing thresholds <25 dB HL at the frequencies of 0.250, 0.500, 1, 2, 3, 4, 6, and 8 kHz. This is the normal level of hearing thresholds). The severity of tinnitus and related distress were measured using the validated questionnaires such as Tinnitus Handicap Inventory (THI) originally developed by Kam et al. [44] and Newman et al. [45]. The characteristics of the participants are presented in Table 1.

This study was approved by medical research ethics committee and institutional review board of Beijing Tongren Hospital, Capital Medical University, Beijing, China, and written informed consent was obtained.

### 2.2. MRI Scanning

Imagines were acquired on a 3.0 T magnetic resonance scanner (General Electric Medical Systems, Milwaukee, WI, USA). The matched eight-channel phased array coil was used with foam padding to reduce head motion and scanner noise. Resting-state fMRI was obtained using an EPI (echo planar imaging) pulse sequence: TR (repetition time)/TE (echo time) = 2000/35 mm, flip angle = 90, field of view = 24 cm × 24 cm, matrix = 64 × 64. Twenty-eight axial slices were obtained with 4 mm thickness and a 1 mm gap. Each fMRI session lasted 400 seconds. A 3-dimensional brain volume (3D-BRAVO) technique was used to acquire high-resolution structural images (TR = 8.8 ms, TE = 3.5 ms, TI = 450 ms, FOV = 24 cm × 24 cm, matrix = 256 × 256, slice thickness  =  1.0 mm without gap, 196 slices, 1 averages). During the scan, subjects were asked to remain motionless, not to think of anything particular during the functional scans.

### 2.3. Data Preprocessing

The preprocessing was carried out by using Data Processing Assistant for Resting-State fMRI (DPARSF) [46] (http://www.restfmri.net/) which is based on Statistical Parametric Mapping (SPM8) (http://www.fil.ion.ucl.ac.uk/spm/) and Resting-State fMRI Data Analysis Toolkit (REST) [47] (http://www.restfmri.net/), including removing the first 20 volumes for the signal equilibrium and participants' adaptation to the scanning noise, slice timing, head motion correction, spatial normalization to the Montreal Neurological Institute (MNI) template (resampling voxel size = 3 mm × 3 mm × 3 mm), linear trend removal, temporally bandpass filtering (0.01–0.08 Hz), and spatially smoothed with a Gaussian kernel of 4 mm full-width at half maximum. Exclusion criteria include a head motion larger than 1.5 mm maximum displacement in any direction or an angular rotation greater than 1.5° throughout the scan.

### 2.4. GM Volume Measurements

Many studies have suggested that regional ALFF results could be influenced by gray matter (GM) volume [48, 49]. We performed a voxel-based morphometry (VBM) analysis to investigate whether a changed functional result is related to changes of GM based on the comparison of volume and concentration of GM between PT patients and normal controls using the 3D-BRAVO sequence. DPARSF [46] was used to analyze the data. The preprocessing protocols including normalization, optional modulation, segmentation, and smoothing are similar to previous studies described [50]. GM intensity maps in the MNI space were obtained by the unified segmentation algorithm. Data were spatially smoothed with 8 mm full width at half maximum Gaussian kernel. Two-sample *t*-tests were performed between the patient and normal control groups. The threshold was set to *P* < 0.01 corrected for multiple comparisons using Monte Carlo simulation. If there is any positive result, the voxel-wise gray matter volume will be taken as covariates in REST calculations. The threshold was also set to *P* < 0.01 corrected for multiple comparisons using Monte Carlo simulation. Results were visualized by the REST Slice Viewer in the REST software.

### 2.5. ALFF Analysis

We applied REST [47] to calculate the ALFF, which is similar to previous studies [51–53]. Briefly, the time courses were first converted to the frequency domain using an FFT (Fast Fourier Transform). The square root of the power spectrum was computed and then averaged across 0.01–0.08 Hz at each voxel. This averaged square root was taken as the ALFF. The ALFF of each voxel was divided by the global mean ALFF value for each subject, resulting in a relative ALFF. The global mean ALFF value was calculated for each participant within a group global mean mask.

### 2.6. Statistical Analysis

Two-sample *t*-tests and Fisher's exact test were used to compare demographic data between two groups. Two-sample *t*-tests were performed to calculate the GM volume and ALFF difference between groups. The ALFF values of all the pulsatile tinnitus patients and healthy controls will be compared by two-sample *t*-tests and the age was included as a covariate. Pearson's correlative analysis was performed using SPSS 12 software (SPSS, Inc., Chicago, IL) to explore the relationships between the THI result, PT duration, and ALFF values of the peak voxels in the patient group. Voxels with a *P* value <0.01 (corrected for multiple comparisons using Monte Carlo simulation) and cluster size >21 voxels were considered to show significant difference between the two groups. Results were visualized by the REST Slice Viewer in the REST software.

## 3. Results 

### 3.1. ALFF Analysis with GM Volume as Covariates

We performed a VBM analysis to reveal the GM changes and its relationship with the ALFF results. The result showed there's no GM volume difference between the two groups after Monte Carlo simulation correction.

### 3.2. ALFF Changes in Pulsatile Tinnitus

**Figure 1:**
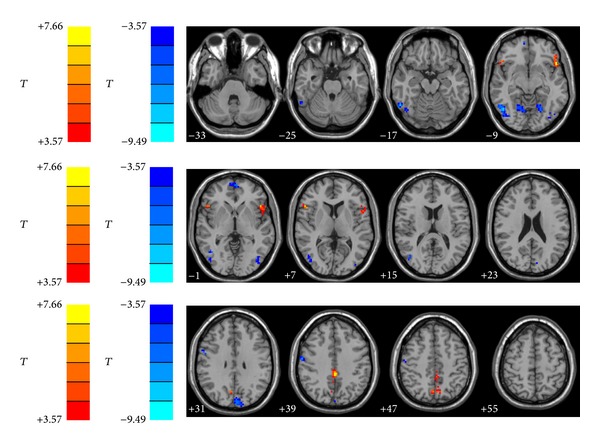
Altered ALFF in PT patients in comparison with controls (two-sample *t*-tests).

Two-sample *t*-tests were performed to assess differences between groups. As shown in Figure 1 and Table 2, after statistically controlling for the age, the bilateral precuneus and inferior frontal gyrus (IFG) showed significantly increased ALFF in the pulsatile tinnitus patients than that in the controls. However, compared to the controls, the left cuneus, right precentral gyrus, and the bilateral middle-inferior occipital gyrus, lingual gyrus, and right superior parietal lobule significantly decreased ALFF in the pulsatile tinnitus patients.

Regions showed that significant increased ALFF values were bilateral precuneus and inferior frontal gyrus. Decreased ALFF values were found in left cuneus, bilateral middle-inferior occipital gyrus, lingual gyrus, right mPFC, right superior parietal lobule, and right precentral gyrus. The left side corresponds to the right hemisphere.

### 3.3. Correlations between THI Score, PT Duration, and ALFF Values

**Figure 2:**
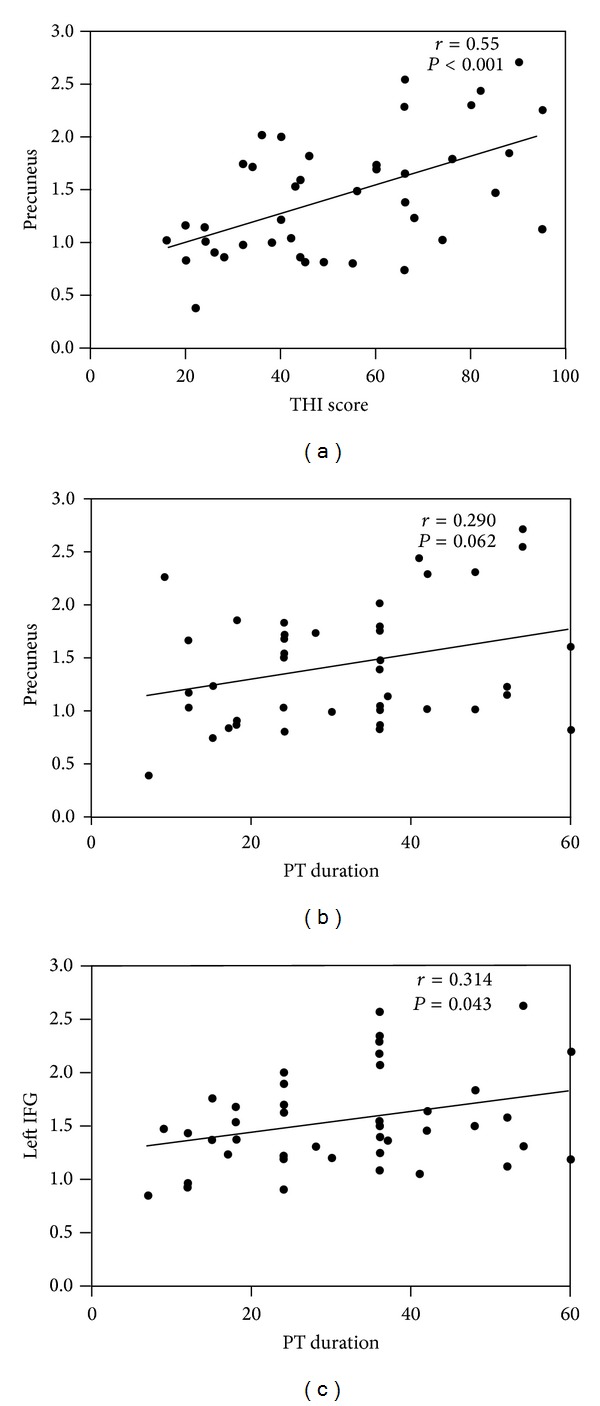
Correlations between THI score, PT duration, and ALFF values in precuneus and left IFG. (a) Correlation between the THI score and ALFF value in the precuneus (*r* = 0.549, *P* < 0.001). (b) Correlation between the PT duration and ALFF value in the precuneus (*r* = 0.290, *P* = 0.062). (c) Correlation between the PT duration and ALFF value in the left IFG (*r* = 0.314, *P* = 0.043). A similar result was also present in the right IFG (*r* = 0.342, *P* = 0.027) (not shown here). *r* = Pearson correlation coefficient. IFG = inferior frontal gyrus.

A significant positive correlation was found between the THI score and ALFF value in the precuneus (*r* = 0.549, *P* < 0.001) (Figure 2(a)). None of the other significant correlations was found.

A correlation trend was also found between the PT duration and ALFF in the precuneus, but it was not statistically significant (*r* = 0.290, *P* = 0.062) (Figure 2(b)). Significant positive correlation was found between the PT duration and left and right IFG (*r* = 0.314, *P* = 0.043; *r* = 0.342, *P* = 0.027, resp.) (Figure 2(c)). None of the other significant correlations was found.

## 4. Discussion

The ALFF could be influenced by regional GM atrophy. The GM atrophy may lead to artificial reduction in regional ALFF results. If there is any abnormality, all the functional results should be adjusted. But the VBM analysis performed in our study showed no significant change in the GM volume of PT patients. Thus, on the basis of our result, the altered ALFF in PT patients in our group are believable. It implies that our results could reflect the changes in intrinsic brain functional activities in the PT patients. Previously, Husain et al. [54] and Mühlau et al. [55] conducted research on patients with nonpulsatile tinnitus (NPT). They concluded that the brain volume could be changed in NPT patients. Apart from the totally different etiology between nonpulsatile and pulsatile tinnitus, there should be some other explanations. The most likely one is the different disease duration. The tinnitus course of those NPT patients was up to 240 months. What is more, a large sample (*n* = 257) makes it more possible to demonstrate statistically significant changed areas of GM in previous NPT patients [22]. However, our study contains relatively fewer patients (42 cases) because of the relatively rare PT. The disease duration of the PT patients (6–60 months) may not be long enough to exert changes in the volume of gray matter. Another possible hypothesis for the diminished gray matter of those NPT patients might be the hearing loss. Husain et al. investigated structural changes related to NPT and hearing loss [54]. They found that they were both gray and white matter changes around the auditory cortex for subjects with hearing loss alone relative to those with tinnitus and those with normal hearing. However, there was no significant gray matter volume changes in NPT patients compared with normal controls. Although they studied relatively fewer NPT patients (only 8 cases) and hearing loss patients (only 7 cases), they concluded that hearing loss, rather than tinnitus itself, had the greatest influence on gray and white matter alterations based on their VBM study [54]. However, the PT patients enrolled in our study all confirmed normal hearing by hearing threshold examination. It is reasonable that no significant changes of brain volume were detected in PT patients. But we still need additional studies to make it clear whether there will be changes in the gray matter in PT patients with longer disease duration.

Abnormal baseline brain activity in several brain areas was found in the PT patients. Increased ALFF brain areas include the bilateral precuneus and bilateral inferior frontal gyrus (IFG). Decreased ALFF brain areas include left cuneus, bilateral middle-inferior occipital gyrus, lingual gyrus, right mPFC, right superior parietal lobule, and right precentral gyrus. Meanwhile, we also focused on the correlation between the ALFF values and clinical data such as THI score and PT duration.

The precuneus is part of the core structure in the limbic system. Parts of the limbic system play a central role in the development of tinnitus [56, 57]. The limbic network were believed to be closely associated with tinnitus distress [38–41, 56, 58, 59]. The precuneus is a highly integrated structure, supposed to be involved in self-consciousness, shifting of attention [60], auditory imagery [61], auditory memory retrieval [62], and memory-related aspects of the tinnitus percept [39, 63]. For those who can cope with the NPT and have only low distress, increased activities were presented in the PCC/precuneus area in previous studies [20, 39]. But there is something we need to pay attention to; these previous studies were based on continuous scalp EEG recordings and sLORETA (low resolution electromagnetic tomography), a tomographic inverse solution imaging technique. The direct relationship between the sLORETA analysis results and the BOLD signal remains unclear. Direct relationship between fMRI signal and EEG activity was reported in a study [64]. There is also some resting-state studies reporting that EEG is not directly linked to the changes in neural activity as measured by BOLD fMRI [65, 66]. On the other hand, Logothetis et al. [67] found that task-induced BOLD signal changes correlated better to local field potential (LFP) than to single unit spiking, indicating that the BOLD response reflects the integration of input and intraneuronal processing. Such a combination is a good way of understanding the nature of LFF [51]. Anyway, one could presume that the fMRI signal change should have a possible correlation with the EEG results but it may not always be accurate. Resting-state PET/SPECT, on the other hand, directly measures the metabolism of different brain areas, reflecting the neural activity in a period of time (while the ALFF measures the deviation of the BOLD signal [51]). It may be a better comparable technique to the LFF according to some authors [68]. Increased activity in precuneus/PCC regions was observed in subjects with major depression (examined by PET (positron emission tomography) scan) [69] and unpleasant music perception (examined by CBF (cerebral blood flow) changes) [70]. In our study, our patients showed increased ALFF value in precuneus. Also we found that ALFF values in precuneus region have significant correlations (*r* = 0.549, *P* < 0.001) with THI score. Thus, even though there was no similar study using ALFF analytic technique to study distressed patients or tinnitus (NPT or PT) patients, the increased ALFF in precuneus of the PT patients is considered to be possibly related to PT awareness as well as the tinnitus related distress. A similar result was also reported in a connectivity analysis of NPT patients using ICA approach based on fMRI (significant correlation between the beta values of PCC/precuneus and THI score, *r* = 0.68, *P* = 0.01) [25]. In fact, nearly 20–60% of tinnitus patients have reported clinical depression [37]. Note also the correlation trend between the activity of precuneus with the disease duration (*r* = 0.290, *P* = 0.062). One can hypothesize that the PT sounds may integrate in the limbic system with time. The altered baseline brain activity in precuneus region should be considered as a kind of modulation secondary to pulsatile tinnitus.

The Precuneus/PCC is also an important part of the default system. This brain system is comprised of Precuneus/PCC, medial prefrontal cortex (MPFC), inferior parietal lobule (IPL), lateral temporal cortex (LTC), parahippocampal gyrus (PHG), and hippocampus. DMN is active with high metabolic rates at rest [71, 72], during self-referential behavior [73], episodic memory processing [74], and so forth but shows divergent fluctuations in spontaneous activities during the task [72, 75]. In our study, the patients maintained high activity in Precuneus/PCC, indicating preservation of normal autobiographical reveries, despite the presence of persistent pulsatile tinnitus. Also, there have been some researches indicating that a positive correlation between the strength of inflow to the temporal cortices and tinnitus-related distress was found in DMN, especially in Precuneus/PCC areas [76]. This corresponds with our correlation analysis between THI score and precuneus.

The role of IFG involved in tinnitus patients is still unclear. In a previous task-fMRI study [77], significant increased signal intensity was found in bilateral IFG after trials with stimulation at the tinnitus frequency. These clusters also showed significant correlation between the tinnitus loudness ratings and the BOLD signal change in that experiment. Even the result may be overestimated due to the task design, the correlation trend is still present. Our results provide further support for abnormal ALFF in bilateral IFG in tinnitus patients; even the loudness of pulsatile tinnitus cannot be measured because of the insufficient measurement and standard. Thus, the increased ALFF in bilateral IFG is considered to be vital areas to identify tinnitus awareness. Moreover, the IFG was critical for response inhibition [78]. The increased ALFF may reflect an inhibitory effort in patients with PT to suppress the sound which is in accordance with the heartbeat. Meanwhile, there are also researches indicating that the brain connectivity is widely disturbed in the tinnitus patients over time [16, 79]. Positive correlations were also found between the ALFF values and disease duration in the left and right IFG (*r* = 0.314, *P* = 0.043; *r* = 0.342, *P* = 0.027, resp.). Thus, we hypothesize that these results are possibly reflecting ongoing changes in neural networks of PT patients. The connectivity research in PT patients is required to confirm the theory above. Early treatment might be essential in ensuring better prognosis.

Decreased ALFF in multiple occipital areas are interesting findings in our research. In a previous PET study, decreased occipital blood flow was reported during auditory tasks in the normal control group without temporal activation [80]. Our results were just similar to this study. In our opinion, the connections between auditory and visual cortex make it possible to alter the brain activity in the occipital areas [27, 81–86]. In blind subjects, the visual cortex was recruited in the context of auditory localization [80, 81]. It was named “auditory occipital activations” (AOAs), which may reflect the visual region processing soon-to-appear objects after sound source stimulation [86]. Contrary to the AOAs, the decreased ALFF in occipital cortex may be caused by neural reorganization in this area. PT patients may need a downregulation adjustment of the AOAs to avoid misinterpreting the sounds around. This could be understood as a kind of “self-protect mechanism” in PT patients. A previous study proved the negative correlations reciprocally characterizing the functional connectivity between auditory and occipital/visual cortex in NPT patients. Could we find similar results in PT patients? What is the difference between these two groups of patients (NPT V.S. PT)? These questions should be answered by more investigations, especially functional connectivity analyses.

The auditory system was involved in patients with nonpulsatile tinnitus [16–27]. However, these areas did not show any difference compared with the normal control group. The possible reason is the totally different sensation and etiology of the pulsatile tinnitus from the nonpulsatile type. The pulsatile tinnitus usually sounds like wind blow, rushing water, or just rumbling in accordance with the heartbeat. But the nonpulsatile tinnitus is a kind of ringing sound with different frequencies. Pulsatile tinnitus is usually caused by vibrations from turbulent blood flow that reaches the cochlea, which often arises from sigmoid sinus diverticulum, atherosclerosis, aneurysm of internal carotid artery, abnormal mastoid emissary vein, dural arteriovenous shunts, paraganglioma, and so forth, [7, 28–30], while the exact etiology of nonpulsatile tinnitus remains unclear. This is the most possible reason that makes the pulsatile tinnitus unique. Thus, it is this particular kind of disease that presents us with results different from the previous ones. We just discuss our findings and try to explore the clinical meanings of the pulsatile tinnitus patients.

## 5. Limitation

There are also some limitations in our research. Firstly, the patients enrolled in this research lack of variance. The etiology of PT is quite a lot. But the common etiology of PT, according to our daily work, was the abnormal blood flow induced by focal defect of mastoid bone shell in the region of the transverse-sigmoid junction. Also, hundreds of patients with this etiology were cured in our hospital, while cured patients with other etiology were not quite common. Thus, the underlying pathology of pulsatile tinnitus and the variability and the possibility that it might influence our results could not be discussed this time. Data of the patients with the most common etiology are easier to acquire and more important to analyze. Patients with different etiology or different kinds of sounds will be enrolled. We firstly investigate the relatively common type and then, we could continue our investigation for more details. Secondly, the neuropsychological tests, such as Mini Mental State Exam (MMSE), Auditory Verbal Learning Test (AVLT), and so forth, were not administrated. But THI is suitable in reflecting the psychological status of the tinnitus patients in our study. We will enroll more tests to evaluate individual's neuropsychological status. Thirdly, subjects in patient group with higher THI score and longer disease duration were not included. We only enrolled patients with disease duration between 6 and 60 months because previous results indicated that the activity and neural network of chronic tinnitus patients are quite different from that of patients with onset disease [16]. Further studies will enroll patients with extensively different disease duration to reveal the changes of brain activity or brain network over time. But the current study was the first to investigate the changes of baseline brain activity in patients with pulsatile tinnitus using resting-state fMRI. Our study is effective in offering information about understanding the changes in brain activity in PT patients. Last but not least, functional connectivity analysis of the tinnitus network is an important part of tinnitus fMRI research. We will apply functional connectivity analyses based on the ALFF results to study the altered brain network of PT patients.

## 6. Conclusion

In summary, multiple altered baseline brain activity areas in patients with pulsatile tinnitus were focused on part of the limbic system, the IFG, and multiple occipital areas. The activity of auditory system was not found to be significantly changed. Results confirmed the disturbances in PT-related neural networks, which may be potentially helpful in understanding the pathophysiology of PT.

## Figures and Tables

**Table 1:** Characteristics of the participants.

	PT (*n* = 42)	HC (*n* = 42)	*P*-value
Gender (male/female)	3/39	3/39	1.000^a^
Age (year)	24–65 (37.2 ± 10.2)	22–64 (37.0 ± 10.0)	0.948^b^
Education (years)	4–19 (12.0 ± 4.3)	5–20 (13.3 ± 4.9)	0.260^b^
Handedness	42 right-handed	42 right-handed	1.000^b^
PT duration (months)	6–60 (31.6 ± 14.4)		
THI score	16–95 (51.5 ± 21.0)		

Data are presented as the range of min–max (mean ± SD); PT: pulsatile tinnitus; HC: healthy controls.

^a^Fisher's exact test.

^b^Two-sample *t*-tests.

**Table 2:** Regions showing significant ALFF differences between PT patients and controls (*P* < 0.01 corrected for multiple comparisons using Monte Carlo simulation).

Brain region	Peak MNI, mm			Peak *T* value	Cluster size, mm^3^
	*x *	*y *	*z *		
Precuneus	0	−36	39	7.4512	94
L inferior frontal gyrus	−48	21	−3	7.6632	63
R inferior frontal gyrus	54	21	6	6.8969	55
R mPFC	9	60	−6	−7.4832	27
L cuneus	−3	−87	30	−6.7625	60
L middle-inferior occipital gyrus	−45	−84	−3	−6.7819	49
R middle-inferior occipital gyrus	57	−57	−8	−9.4918	94
L lingual gyrus	−21	−63	−9	−6.7887	33
R lingual gyrus	21	−63	−9	−6.1621	44
R precentral gyrus	54	−27	21	−7.3635	38
R superior parietal lobule	24	−51	63	−7.3521	24

R: right; L: left; MNI: Montreal Neurological Institute. The threshold was set *P* < 0.01 corrected for multiple comparisons using Monte Carlo simulation. mPFC: ventromedial prefrontal cortex.
